# Shame on You! When and Why Failure-Induced Shame Impedes Employees’ Learning From Failure in the Chinese Context

**DOI:** 10.3389/fpsyg.2021.725277

**Published:** 2021-10-06

**Authors:** Wenzhou Wang, Shanghao Song, Jiaqi Wang, Qi Liu, Lishi Huang, Xiaoxuan Chen

**Affiliations:** Department of Human Resource Management, Business School, Beijing Normal University, Beijing, China

**Keywords:** shame, project commitment, restoration orientation, loss orientation, learning from failure

## Abstract

The emotional experience brought about by failure, especially the important roles of negative emotions in learning behavior after failure, has received increasingly more attention from organization management scholars. Research on the impact of employees’ sense of failure-induced shame is still controversial. Based on the Chinese context, according to the process model of emotion regulation theory, we have studied the influence of failure-induced shame on employees’ learning from failure and the conditions that have boundary effects on this process. Through a questionnaire analysis of 776 samples from Chinese high-tech enterprises, the results show the following: (1) shame has a negative relationship with learning from failure (2) project commitment alleviates the negative relationship between shame and learning from failure, and (3) restoration orientation alleviates the negative relationship between shame and learning from failure while loss orientation cannot. Our results further enrich the research on negative emotions related to failure and provide a theoretical basis for the failure management of Chinese companies.

## Introduction

In a complex and ever-changing business environment, it is common for enterprises to encounter project failure. Studies have shown that failure experiences can encourage organization members to find effective problem solutions and challenge old concepts to innovate (Ingram and Baum, 1997). Learning from failure enables employees to gain experiences and lessons from project failure, adjust cognitive and behavioral patterns, and then reduce the possibility of similar failures in the future (Shepherd et al., 2011). Although learning from failure has many benefits, there are many factors affecting the process of employees’ learning from failure in real situations, and negative emotion is one of the important aspects (Shepherd and Cardon, 2009; Zhao, 2011). Negative emotions refer to individuals’ psychological reactions when they fail in an organization or a new project (Smith and Mcelwee, 2011), affecting employees’ sense of failure, failure attribution, and determination to continue to work (Shepherd and Cardon, 2009), which affect the process of learning from failure in both aspects of motivation and ability.

After the failure of a project, the most common emotional responses are guilt and shame (Bohns and Flynn, 2013). These two emotional responses are similar but different. Guilt is a type of adaptable emotion that can stimulate constructive behaviors, while shame is a nonadaptable emotion that can bring more destructive behaviors, such as withdrawal, enmity, and resistance (Tangney, 1991; Tangney et al., 1992; Tangney and Dearing, 2002). Although both of these emotions are typical negative emotions generated after failure, the theoretical concern about shame is much less than guilt. Previous studies have shown that the source of shame is employees’ internal and stable attribution of failures, which leads to employees’ negative feelings about their overall self (i.e., “who I am”; Cohen et al., 2011). It can inhibit employees’ motivation to correct errors and reduce their confidence in making subsequent improvements (Bohns and Flynn, 2013). In the field of learning from failure, Bohns and Flynn (2013) showed that shame is more harmful to employees’ learning from failure behavior than guilt through theoretical deduction. However, this study did not consider the differences between Eastern and Western cultural backgrounds nor did it propose possible boundary variables on how to alleviate the negative impact of shame. Mianzi, as a typical Eastern culture characteristic, is a social psychological construction rooted in culture. Mianzi is increasingly valued by scholars in management research, but it is still lacking in the field of learning from failure research. If cultural differences are considered, the exploration of nonadaptive emotions such as shame can further explain the factors hindering employees from learning from failure. Therefore, we will further explore the relationship between shame and learning from failure based on the cultural difference.

Shame can lead employees to fall into a vicious circle of negative behavior responses and negative emotions (Lewis, 1987; Bohns and Flynn, 2013). Whether there are some boundary variables that can break this cycle, reduce the adverse effects of shame, and even have a positive impact on it has become an urgent problem to be solved. According to Gross’s process model of emotional regulation, both cognition and behavioral responses will affect the occurrence and experience of emotions, and these emotional regulation strategies will affect subsequent cognitive processes and behaviors (Gross and John, 2003). Therefore, from the two aspects of cognition and emotions, this paper takes the negative emotion coping orientations and employees’ project commitment as the moderating variables to explore their roles in the adjustment of negative emotions and subsequent learning from failure behaviors. First, employees can alleviate the shame caused by failures by adapting to negative emotions (Stroebe and Schut, 1999). Shepherd (2003) proposed a dual-process model based on grief theory for individuals to cope with negative emotions (Shepherd, 2003). According to the model, there are two different ways for individuals to cope with negative emotions after failure, which are loss orientation (i.e., a problem-focused coping orientation) and restoration orientation (i.e., an emotion-focused coping orientation). The research suggests that individuals can adjust their emotions by making sense of failure through loss orientation and speed up their emotional recovery by shifting their attention through restoration orientation, which can make the memory related to the original failure gradually disappear; furthermore, both loss orientation and restoration orientation can reduce the negative effects of negative emotions on individuals (Shepherd, 2003).

In addition, the cognition of the goal and value of a project will also affect the emotional and behavioral results after failure. Project commitment, which is an individual’s recognition of the value of a project and their desire to realize the project, affects individuals’ enthusiasm and initiative in work and learning (Wang et al., 2018). Individuals with a high level of project commitment take various measures to achieve project objectives, which enables them to regard failure experiences as an important source of feedback on behavior and learning (Hoegl et al., 2004; Wang et al., 2018). As a result, they can change their perception of failure and learn from it through positive actions and cognitive adjustment, even if they experience shame after failure. Therefore, we assume that negative emotion coping orientation and project commitment may have moderating effects on the relationship between shame and learning from failure.

**Figure 1:**
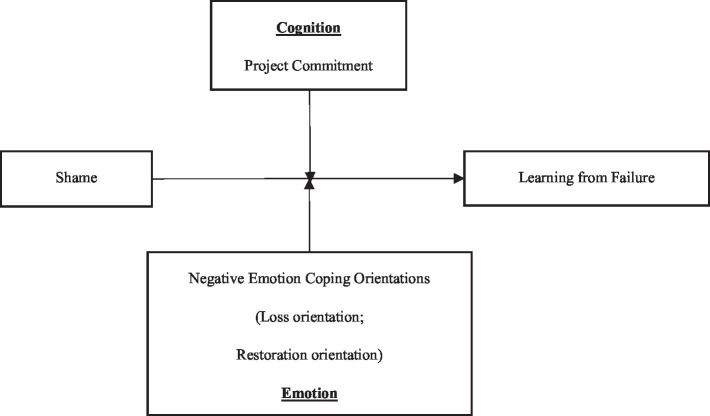
Theoretical framework.

Therefore, we construct a theoretical framework, as shown in Figure 1, to explore the relationship between failure-induced shame and learning from failure in the context of failure. According to the process model of emotional regulation, this study addresses the limitations of previous studies, expands the cultural background of the results of shame-related effects and provides evidence to support subsequent studies.

## Literature Review and Hypotheses

Shame is a type of self-awareness of the negative emotional reactions that often occur in moral situations (e.g., infractions) or nonmoral situations (e.g., project failures; Weiner, 1986). It is described as a painful experience of the entire self when individuals attribute their negative behavior to their own lack of ability (Weiner, 1986). In the business environment, project failure means that the final result of a project in which an individual has invested much effort does not meet their expectations (McGrath, 1999). Therefore, according to the source of shame, many scholars have stated that individuals often experience shame after project failure.

Different scholars have proposed different opinions on the influence of shame on individuals. Most scholars believe that shame has a negative impact on individuals. In the face of a specific event, individuals will experience a series of physiological and physical reactions (e.g., rapid heartbeat, trembling hands and feet; Smith et al., 2002). These embarrassing situations usually make individuals hide their bad selves by avoiding behaviors so as to avoid the destruction of their self-image and the exposure of self-defects (Tangney et al., 1996). However, some scholars propose a different point of view, arguing that shame can promote individuals’ self-image repair motivation, which is further related to their self-compensation behavior and has an impact on the strength of the compensation motivation in a specific situation (Hooge et al., 2007). Based on these discussions, we speculate that whether shame leads to positive or negative behavior may be related to specific event situations.

Guilt, another emotional experience of failure, is often compared with shame. Regarding the influence of guilt on individuals, most scholars hold a positive attitude, believing that guilt usually motivates individuals to compensate, such as by apologizing (Nelissen and Zeelenberg, 2009). In addition, some scholars have found a positive relationship between guilt and learning from failure through empirical studies (Bohns and Flynn, 2013). However, no researchers have examined the relationship between shame and learning from failure. Compared with guilt, shame usually shows more negative mental states (e.g., helpless, powerless, feeling exposed) and behavioral motivations (e.g., avoid, hide, withdraw, escape, isolate, a desire to disappear; Kim et al., 2011). And will these negative states be more obvious in failure context? Moreover, due to cultural differences, individuals in Eastern and Western cultures will have different emotional and cognitive responses to shame generated by negative events (You, 1997). Chinese people pay more attention to “mianzi” (also called “lian” or “face”), which leads to Chinese people paying more attention to self-avoidance when they are ashamed and avoid “losing face” because of revealing their negative image (Bedford and Hwang, 2003). Therefore, cultural differences also need to be considered when exploring the role of shame. In fact, some scholars have combined face to learning from failure-related research (e.g., Wang et al., 2019). However, there is still a lack of further description of its specific mechanism. Based on these considerations, we will shift our perspective to the negative situation of project failure and explore the specific mechanism of the impact of shame on learning from failure in the context of Eastern culture.

In response to the possible adverse effects of shame on individuals, we further propose the following questions: Are there possible cognitive or emotional factors that may affect this process? What factors can weaken the possible adverse effects of shame? First, in the face of negative emotions brought by failure, the degree of healing and the recovery effect of trauma depend on the negative emotion coping methods adopted by individuals. Shepherd (2003) proposed a grief recovery model based on the theory of individual coping with loss and argued that individuals usually handle the negative emotions brought about by events through restoration orientation and loss orientation. Restoration orientation refers to “the suppression of feelings of loss and proactiveness toward secondary sources of stress that arise from a loss” (Stroebe and Schut, 1999). By shifting attention, individuals who adopt restoration orientation can effectively shift the cognitive attention objects brought about by negative emotions, which can relieve the adverse psychological state brought about by negative emotions and help individuals escape the negative psychological experience brought about by shame. Loss orientation refers to “the concentration on, and dealing with processing of some aspect of the loss experience itself” (Stroebe and Schut, 1999). Since individuals who adopt loss orientation pay more attention to the solution of events, they usually take some positive and active actions (such as communicating with colleagues around them), which can help them learn experience and knowledge from failures more quickly (Shepherd et al., 2011). At the same time, Shepherd (2003) also proposed that if an individual can alternately adopt loss orientation and restoration orientation, it is called oscillation orientation. As the oscillation orientation is based on loss orientation and restoration orientation, we did not consider the effect of oscillation orientation and separately considers the role of loss orientation and restoration orientation, in order to further clarify the different roles of two negative emotion coping orientations in the process of regulating emotions. Therefore, we hypothesized that two negative emotion coping orientations could effectively help employees alleviate the negative effects of shame through emotional recovery or event resolution.

In addition to the positive impact of negative emotion coping orientations, Phelps (2006) states that individual cognition of events also plays an important role in the relationship between emotion and behavior. Project commitment refers to individuals’ affective cognitive trait toward a project, which is defined as “one’s belief in the goals and values of a project, and desire to participate in and be a member of the project” (Mowday et al., 1979). As the emotional bond between organizational employees and projects and organizational goals, project commitment has received attention from organizational management scholars as a personality trait. Most previous studies on project commitment by scholars took it as an antecedent variable and studied its possible positive results, such as improving project performance (Gulzar et al., 2012) and promoting learning from failure (Wang et al., 2018). However, whether project commitment plays a positive role in the relationship between emotion and behavior, especially in the context of failure, has not been explored by scholars. We used project commitment as a moderating variable to compensate for the deficiencies in existing studies.

### Shame and Learning From Failure

Shame is a self-conscious emotion generated by self-reflection and self-evaluation when a person’s failures or shortcomings are displayed publicly, and it is often accompanied by fear of rejection or abandonment by the group (Augsburger, 1986). Shame results in employees’ negative self-evaluation and withdrawal tendencies, which leads to poor psychological function and reduces individuals’ willingness and effects of learning (McGregor and Elliot, 2005). Therefore, the shame of failure may not be conducive to learning from failure.

First, shame will lead to negative self-evaluations, which can lower employees’ self-efficacy. Thus, shame has a negative impact on learning intention and efficiency (Arenas et al., 2006; Baldwin et al., 2006). Prior research has shown that shame triggered by specific events is strongly associated with negative psychological symptoms such as low self-esteem, personal distress, neuroticism, and low self-compassion (Tangney and Dearing, 2002; Stuewig and McCloskey, 2005; Tangney et al., 2007), which reduces employee self-efficacy (Cohen et al., 2011) and makes employees’ psychological function decline. Therefore, it is not conducive to increasing employees’ learning motivation, gaining more knowledge from failure, and improving their work skills. Furthermore, shame causes people to attribute errors to their core competencies, which can also undermine their motivation to correct mistakes and reduce their self-confidence to make subsequent improvements (Bohns and Flynn, 2013). The shame of failure results in employees’ avoidance of handling the consequences of their own misbehavior (Tangney and Dearing, 2002), which further leads to a decrease in work willingness, the analysis and exploration of failure events, and the chance of acquiring knowledge from failure experiences.

Second, shame can bring a feeling of losing face. Consequently, employees tend to be evasive about failure events and avoid public attention, which prevents people from communicating about failure (Bohns and Flynn, 2013; Ghorbani et al., 2013). Shame will also hinder the acquisition and feedback of failure information and thus is not conducive to effective learning from failure. Shame in Chinese culture, unlike that in Western culture, is more concerned with morality (Bedford and Hwang, 2003). Project failure meant that a person had not fulfilled his duties. This will make him lose status, and it also means that he needs to bear moral condemnation in Confucian culture (Hwang, 2001). As a result, individuals who feel ashamed often feel “a fear of losing face,” that is, a sense of threat to their status (Chi et al., 2015). Therefore, for the sake of “saving face,” it is very difficult for failed employees to face their leaders and other colleagues, and they tend to belittle or condemn themselves, believing themselves to be fundamentally flawed. In addition, they are afraid of being despised; and as a result, they tend to avoid public attention and escape the consequences of their failures (Eisenberg, 2000; Tangney and Dearing, 2002), which prevents employees from reporting and discussing their faults with others. Moreover, shame even causes employees to cover up their mistakes, thus reducing the opportunities for employees to learn from their failures. In addition, people who feel shame are more likely to develop feelings of resentment, doubt, and blame, which makes it harder for them to seek help from colleagues and consequently decreases their productivity in handling and learning from failures (Tangney, 1995).

In addition, shame will add cognitive stress to employees, which leads to distraction and diffuses the individual resources devoted to learning failure, thus having a negative impact on the efficiency and quality of learning from failure (Lewis, 1987; Shepherd et al., 2011). First, shame is highly correlated with personal pain responses such as self-loathing and self-deprecation (Eisenberg, 2000), which interfere with employees’ concentration on failure and thus are not conducive to reflection and learning from failure. Second, employees with a sense of shame can become hostile and lash out (Tangney et al., 2007; Stuewig et al., 2010), after which they will realize that the behavior is inappropriate, and their negative self-perception will be aggravated (Lewis, 1987). This creates an additional cognitive burden for employees and therefore distracts employees and affects their identification and analysis of the cause of failure. Thus, it is disadvantageous to form an objective and reasonable explanation for the failure event and gain experience from failure.

Instead, when employees have a lower level of shame after a failure event, they bear less psychological and cognitive stress and have less resistance to failure. This results in more willingness to report failure events, share each other’s views, and actively seek the causes and solutions of failure. Consequently, employees will be willing to learn from failure and do well with it. Thus, we assume the following:

*Hypothesis 1*: Failure-induced shame has a negative effect on employees’ learning from project failures.

### The Moderating Role of Project Commitment

As an emotional bond between employees and projects and organizational goals, project commitment has attracted the attention of organizational management scholars. Many scholars believe that individuals with a sense of project commitment usually engage in active learning and other positive behaviors to achieve project goals (Hoegl et al., 2004). Therefore, individuals with project commitment can change their perception of failure and learn from it through positive action and cognitive adjustment, even if they experience shame after failure.

First, employees with a high sense of project commitment often contribute to the team or the organization because of their high sense of identity and responsibility to the organization. They take delight to do things like sharing knowledge for the organization and actively participate in team activities (McDonough, 2000; Jarvenpaa and Staples, 2001). These organizational activities can effectively help employees who are immersed in the shadow of failure to enter the learning status quickly, face failure, and reduce the fear of failure caused by shame (Hislop, 2003).

Second, when an organization is faced with failure, employees with a high sense of project commitment have the motivation to learn energetically and to help others learn from failure and simultaneously facilitate project goals through communication and sharing knowledge with colleagues (Jones et al., 2003; Yang, 2007; Terzieva and Morabito, 2016). As a result, employees with project commitment have more opportunities to actively communicate with others, and they can access information about failure events more quickly, which can help employees adjust their behavior in time to mitigate adverse effects.

In addition, employees with a high sense of project commitment often have a stronger incentive to reflect on the problems they face in advancing projects in order to achieve better performance and cut losses by adjusting their behavior in time (Wang et al., 2018). While failure can have negative consequences, it can also have many benefits, such as providing valuable information such as our weaknesses in how to handle problems (Corbett et al., 2007). As a result, employees with a sense of project commitment will take the initiative to reflect on failure events, pay more attention to failure events, and try their best to cushion the negative impacts of shame on cognition and attention.

Conversely, it is difficult for employees with low project commitment to maintain the belief that they should contribute to the team and the organization. When faced with project failure, they cannot take the initiative to communicate with colleagues so as to engage in reflection on the failure and learn from it. It is harder for them to get through the shame they feel. Thus, we assume the following:

*Hypothesis 2*: Employees with strong project commitment can mitigate the negative relationship between failure-induced shame and learning from failure.

### The Moderating Role of Negative Emotion Coping Orientations

Negative emotion coping orientations refer to the ways individuals handle grief caused by failure, and they include two different coping orientations: loss orientation and restoration orientation (Shepherd, 2003). Both of them play important roles in the process of emotional recovery after failure. Individuals quickly eliminate negative events by focusing on emotional recovery or focusing on event resolution so as to improve their ability to learn from failure (Shepherd, 2003).

Restoration orientation can be divided into two aspects: One is distraction from failure (i.e., avoidance restoration orientation), and the other is to proactively address secondary stressors (such as “What is my role in the organization after a project failure?” and “How can I fit into my new team?”; i.e., proactiveness restoration orientation; Shepherd et al., 2011). Restoration orientation influences employees’ scanning and interpretation of failure information (Yu et al., 2018) and promotes employees’ communication on failure events (Cannon and Edmondson, 2001). For this reason, it can alleviate the effect of shame on learning from failure. Employees using restoration orientation can purposefully distract themselves from loss-related thoughts and allow loss-related memories to fade (Shepherd, 2003), which relieves fear associated with shame that interferes with an employee’s attention (Mogg et al., 1990). Furthermore, it improves employees’ information processing abilities and facilitates the exploration and explanation of failure (Nabi, 1999). Furthermore, actively addressing problems other than failure reduces employees’ feelings of failure, making it easier for employees to access information about failure and adapt to changing circumstances (Cope, 2011). It is also helpful for employees to scan the information on failure so as to have a more comprehensive explanation of failure and promote learning from failure.

Another type of negative emotion coping orientation is loss orientation, and loss orientation enables employees to construct new meanings of failure (Shepherd, 2003). By reflecting on failure events, employees can re-examine past assumptions and change their understanding of failure events (Mezirow, 1991). First, loss orientation affects employees’ ability to scan and interpret failure information. Loss orientation allows employees to directly confront failure and events that lead to failure; therefore, they are able to gather comprehensive information about failure (Shepherd et al., 2011). This is conducive to the formation of a comprehensive and objective interpretation of the failure and leads to the correct attribution of failure. Second, loss orientation will allow employees to give meaning to failure, which is beneficial to improving the willingness to learn from failure. The shame of failure leads to a negative self-evaluation (Baldwin et al., 2006), so employees tend to have a negative reaction to failure, which leads to cognitive biases. Therefore, employees may cover up failures by means of direct manipulation (McGrath, 1999), which has an adverse effect on the objective analysis of failure. Loss orientation makes employees not only face the loss of failure but also gain from the failure (Archer, 1999). For example, feedback information is available, which forces individuals to make changes to improve themselves. In conclusion, loss orientation can alleviate the negative effect of shame on learning from failure (Lewis, 1987; Retzinger and Scheff, 2000; Gausel, 2012).

In contrast, if employees do not adopt restoration orientation or loss orientation when addressing negative emotions, it is difficult for them to adjust their mental state and reduce the effect of shame on attention. It does not help employees re-examine the problems in the failure event but increases the negative impact of shame on learning. Thus, we hypothesize the following:

*Hypothesis 3a*: Employees with a strong restoration orientation can mitigate the negative relationship between failure-induced shame and learning from failure.

*Hypothesis 3b*: Employees with a strong loss orientation can mitigate the negative relationship between failure-induced shame and learning from failure.

## Materials and Methods

### Participants and Procedure

High-tech companies involve more creative work, and they are more likely to fail. Therefore, the ability to learn from failure is essential in the rapid development of high-tech industries. Therefore, this study took the members of high-tech companies (the annual sales of high-tech products or services accounted for 60% of its total sales and at least 10% of its employees are R&D personnel) as research objects. We randomly selected 400 companies from the list of high-tech companies reported in Beijing and phoned them to invite them to participate in our research. When communicating with the companies, we explained the purpose of the research, emphasized the confidentiality of the data, and promised to share the research results with the company leaders. For companies that agreed to participate in the survey, with the help of an internal coordinator appointed by the CEO, the research assistant, after identifying participants, distributed the questionnaire and asked team members to complete the questionnaire before regular team meetings. For the absentees, research assistants obtained their contact information from the internal coordinator and ensured that they returned the questionnaire through follow-up communication. In addition, in order to improve the response rate of the questionnaire, we asked the CEO to leave an endorsement and distribute a small gift with the questionnaire.

Finally, we obtained completed questionnaires from the full sample of 776 participants. The average age of these participants was 31.65 (ranging from 20 to 56years, SD=5.497), of which 77.1% were men, and approximately 92.4% of the participants had a bachelor’s degree or above.

### Measures

All items in the questionnaire came from extant research. To ensure the accuracy of the translation, we used the back translation method (Brislin, 1980) with two independent professional translators to first translate questions from English to Chinese and then back translate them into English. In addition, we defined project failure as the termination of a project because the plan did not achieve its objectives (Shepherd et al., 2011), and this definition was given in the introduction section at the beginning of the questionnaire to ensure that participants could better understand the purpose of the research.

#### Shame

We used a five-item scale (6-point Likert scale) to measure shame (Scherer and Glueckauf, 2005). Sample items included “fear of losing face” and “embarrassed.” Cronbach’s alpha coefficient of the scale was 0.929.

#### Negative Emotion Coping Orientation

We used the six-item scale developed by Shepherd et al. (2011) to measure negative emotion coping orientation. Sample items of restoration orientation include “I intentionally divert my attention, not thinking about the problem of the project failure” and “After the project fails, I try to sort out my thoughts.” Cronbach’s alpha coefficient of the scale was 0.605. Sample items of loss orientation include “I worked with my colleagues to find the cause of the failure” and “I worked hard to overcome the negative emotions associated with the failure of the project.” Cronbach’s alpha coefficient of the scale was 0.702.

#### Project Commitment

We used a five-item scale (6-point Likert scale) to measure project commitment (Hoegl et al., 2004). Sample items included “I feel I have a responsibility to achieve the goals of the project” and “I am highly invested in the project and the project team.” Cronbach’s alpha coefficient of the scale was 0.863.

#### Learning From Failure

We used an eight-item scale (6-point Likert scale) to measure learning from failure (Shepherd et al., 2011). Sample items included “I can manage a project more effectively” and “I can find possible problems in a new project timelier.” Cronbach’s alpha coefficient of the scale was 0.909.

#### Control Variables

We controlled not only demographic variables, such as sex (male defined as 1 and female defined as 2), age, education level (including high school, technical secondary school, college, undergraduate, master’s, doctoral and others), and tenure in the firm and project team, but also oscillation orientation, which is parallel to restoration and loss orientation (Shepherd et al., 2011). Additionally, we controlled for fear of losing face, which is a contextual variable often used in Chinese culture. Previous research has confirmed that fear of losing face will have an impact on an individual’s cognition and behavior (i.e., learning from failure; Wang et al., 2019).

##### Oscillation Orientation

We used a three-item scale developed by Shepherd et al. (2011) to measure oscillation orientation. Sample items include “After giving my emotions a rest, I confront my negative feelings arising from the project’s failure” and “After thinking about the failure for a period of time, I try not to think about it as much as possible.” Response options ranged from 1 (strongly disagree) to 6 (strongly agree). Cronbach’s alpha coefficient of the scale was 0.582.

##### Fear of Face Loss

We used a five-item scale developed by Zane and Yeh, (2002) to measure fear of losing face. Sample items include “I don’t criticize others because it might embarrass them” and “Before speaking or doing something, I will think carefully and avoid making mistakes.” Response options ranged from 1 (strongly disagree) to 6 (strongly agree). Cronbach’s alpha coefficient of the scale was 0.577.

## Hypothesis Testing

We used Amos 24.0 for confirmatory factor analysis (CFA) to check the validity of the model and SPSS 25.0 for correlation analysis and multiple regression analysis to test the hypothesis. Our data were collected at the same time, which may lead to common method bias (CMB). Following Podsakoff et al. (2003), we further conducted Harman’s single-factor test. The results of CFA and Harman’s single-factor test together indicate that CMB may not be a substantial problem in this study.

### Confirmatory Factor Analysis

In order to test the degree of model fit, we first conducted CFA. As shown in Table 1, the fitting index of our theoretical model (six-factor model) is the best compared with other models (CMIN/DF=2.878, IFI=0.918, TLI=0.907, CFI=0.918, RMSEA=0.049). Therefore, the model of this study is valid.

**Table 1:** Confirmatory factor analysis.

Model	CMIN	DF	CMIN/DF	IFI	TLI	CFI	RMSEA
Six-factor Model	1784.091	620	2.878	0.918	0.907	0.918	0.049
(Shame, LoF, ProC, LO, RO, OO, LFF)							
Five-factor Model	2282.912	619	3.688	0.898	0.889	0.897	0.058
(Shame+LoF, ProC, LO+RO, OO, LFF)							
Four-factor Model	2624.121	618	4.246	0.889	0.877	0.887	0.062
(Shame+LoF, ProC, LO+RO+OO, LFF)							
Three-factor Model	3592.842	621	5.786	0.829	0.806	0.826	0.089
(Shame+LoF+ProC, LO+RO+OO, LFF)							
Two-factor Model	4774.343	624	7.651	0.714	0.707	0.716	0.106
(Shame+LoF+ProC+LO+RO+OO, LFF)							
Single-factor Model	5620.574	621	9.051	0.695	0.687	0.693	0.110
(Shame+LoF+ProC+LO+RO+OO+LFF)							

*LoF, Fear of face loss; ProC, Project Commitment; LO, Loss Orientation; RO, Restoration Orientation; OO, Oscillation Orientation; LLF, Learning from failure; N=776*.

### Descriptive Statistics and Correlation Coefficient Test

The descriptive statistics and correlation coefficients in Table 2 include the means, standard deviations, Cronbach’s alpha coefficients of the variables, and the correlation between variables. As seen from Table 2, there is a significant negative relationship between shame and learning from failure (*b*=−0.098, *p*<0.01), which supports hypothesis 1.

**Table 2:** Means, standard deviation, and descriptive statistics.

	M	SD	1	2	3	4	5	6	7	8	9	10	11	12
1 Sex	1.229	0.420												
2 Age	31.648	5.497	−0.040											
3 Education	4.360	0.677	0.000	0.190^**^										
4Years in the Firm	1.764	0.726	−0.008	0.685^**^	0.187^**^									
5Years in the Team	1.464	0.680	−0.040	0.490^**^	0.214^**^	0.761^**^								
6 Fear of face loss	3.682	0.688	0.027	−0.037	0.015	0.039	0.024	(0.577)						
7 Shame	2.498	1.178	−0.148^**^	0.021	−0.109^**^	−0.018	−0.023	0.109^**^	(0.929)					
8 Project Commitment	4.443	0.860	0.016	0.007	−0.074^*^	0.002	0.051	0.037	−0.041	(0.863)				
9 Loss Orientation	3.911	0.733	0.064	0.010	−0.140^**^	0.002	0.040	0.095^**^	0.137^**^	0.393^**^	(0.702)			
10 Restoration Orientation	3.872	0.668	0.043	−0.001	−0.068	0.041	0.051	0.165^**^	0.109^**^	0.275^**^	0.491^**^	(0.605)		
11 Oscillation Orientation	4.008	0.862	0.079^*^	−0.001	−0.066	0.013	−0.002	0.149^**^	0.049	0.293^**^	0.420^**^	0.656^**^	(0.582)	
12 Learning from Failure	4.580	0.839	0.054	−0.024	−0.040	0.048	0.056	0.024	−0.098^**^	0.477^**^	0.417^**^	0.379^**^	0.417^**^	(0.909)

* *p<0.05*;

** *p<0.01; N=776*.

### Hypothesis Testing

We adopt the stepwise linear regression method to further test the relationships between the variables. Specifically, we use learning from failure as a dependent variable to conduct a regression in six steps (Table 3). When examining the moderating effects of project commitment and negative emotion coping orientation, we standardized the independent variable (shame) and the moderating variables (project commitment and negative emotion coping orientation) and constructed the interaction term (Aiken and West, 1991) in order to reduce the impact of collinearity. The results are shown in Table 3.

**Table 3:** Results of regression analysis.

Variables	Learning from Failure						
	Model 1	Model 2	Model 3	Model 4	Model 5	Model 6	Model 7
1 Sex	0.108	0.078	0.000	−0.002	−0.005	0.003	−0.001
2 Education	0.008	0.008	0.001	−0.004	−0.005	0.000	−0.001
3 Age	−0.050	−0.065	0.024	0.021	0.026	0.032	0.028
4Years in the Firm	0.009	0.009	0.007	0.006	0.006	0.005	0.005
5Years in the Team	0.008	0.008	0.001	0.001	−0.001	0.001	0.000
6 Fear of face loss	0.027	0.041	−0.046	−0.049	−0.053	−0.052	−0.055
8 Shame		−0.072^**^	−0.088^***^	−0.098^***^	−0.095^***^	−0.097^***^	−0.103^***^
9 Project Commitment			0.310^***^	0.300^***^	0.305^***^	0.302^***^	0.296^***^
10 Loss Orientation			0.216^***^	0.219^***^	0.222^***^	0.225^***^	0.226^***^
11 Restoration Orientation			0.111^*^	0.113^*^	0.125^*^	0.126^*^	0.127^*^
12 Oscillation Orientation			0.193^***^	0.197^***^	0.192^***^	0.200^***^	0.201^***^
13 Shame×Project Commitment				0.076^**^			0.050^*^
14 Shame×Loss Orientation					0.070^**^		0.022
15 Shame×Restoration Orientation						0.088^***^	0.063^*^
*R^2^*	0.009	0.018	0.359	0.368	0.366	0.371	0.375
*△R^2^*		0.009	0.341	0.009	0.007	0.012	0.016
*F*	1.122	2.05	36.801	36.878	36.586	37.349	32.566
*P*	0.347	0.047	0.001	0	0	0	0

* *p<0.05*;

** *p<0.01*;

*** *p<0.001; N=776*.

Hypothesis 1 states that shame will impede an individual’s learning from failure behavior. Table 3 shows that there is indeed a significant negative relationship between shame and learning from failure (*b*=−0.072, *p*<0.01, Model 2). Therefore, hypothesis 1 is supported. Hypothesis 2 and hypothesis 3 address the moderating effects of project commitment and negative emotion coping orientation (loss orientation and restoration orientation), respectively. As shown in Table 3, when the three moderators are put into the model separately, there is a significant positive relationship between the interaction item of shame and project commitment and learning from failure (*b*=0.076, *p*<0.01, Model 4). There is also a significant positive relationship between the interaction item of shame and loss orientation and learning from failure (*b*=0.070, *p*<0.01, Model 5). Furthermore, there was a significant positive relationship between the interaction item of shame and restoration orientation and learning from failure (*b*=0.088, *p*<0.001, Model 6). However, when the three moderators were put into the model at the same time, the moderating effects of project commitment and restoration orientation are still significant (b_shame × project commitment_=0.050, *p*<0.05; and b_shame × restoration orientation_=0.063, *p*<0.05, Model 7), indicating that the moderating effect of the two moderators is stable; however, the moderating effect of loss orientation becomes nonsignificant (b_shame × loss orientation_=0.022, *p*>0.05, Model 7). Therefore, the moderating effects of project commitment and restoration orientation are supported while the moderating effect of loss orientation is not supported by the data.

**Figure 2:**
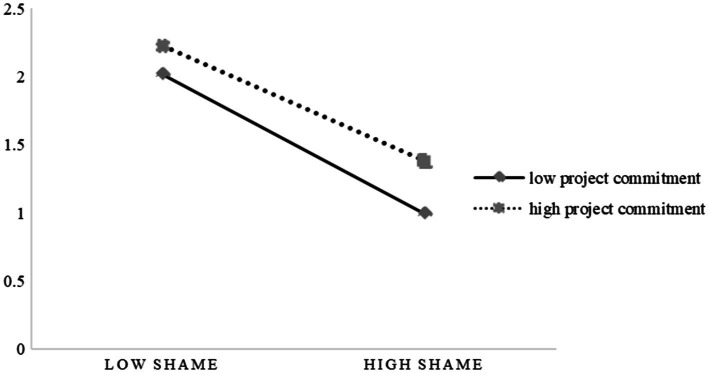
The moderating role of project commitment.

**Figure 3:**
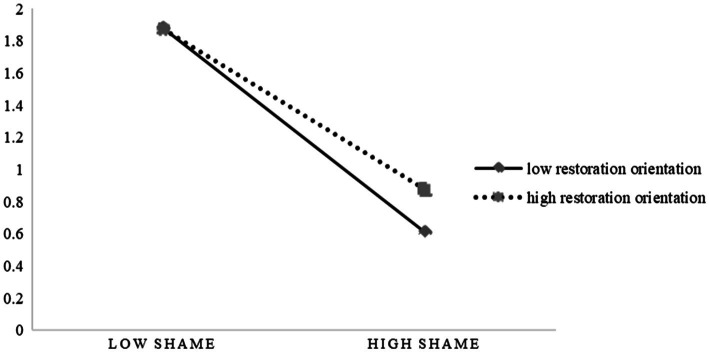
The moderating role of restoration orientation.

In order to better explain the moderating effects of project commitment and restoration orientation, we take the data higher than (or lower than) the average value of a standard deviation as the value of the moderating variable at a high (or low) level (the average value of the variables is 0, and the standard deviation is 1) and draw the relevant schematic diagrams, as shown in Figures 2, 3.

## Discussion

Project failures are common in the workplace. They have not only visible financial costs and interruptions in project progress but also consequential psychological costs to employees (e.g., a range of negative emotions). In this paper, we investigate the influence mechanism of individual negative emotions caused by failure on subsequent learning from failure behavior and identify the affective and cognitive factors that may affect this relationship as moderators. Specifically, this paper constructs a theoretical framework, taking feelings of shame after failure as an antecedent variable and negative emotion coping orientation and project commitment as moderators, to explain how and to what extent negative emotions affect people’s learning from failure events. Through the empirical analysis of questionnaire data from high-tech enterprises in Beijing, we draw the following conclusions: (1) Failure-induced shame has a negative relationship with employees’ learning from failure; (2) restoration orientation negatively moderates the relationship between failure-induced shame and learning from failure, and the higher the restoration orientation level is, the weaker the negative relationship is, but the moderating effect of loss orientation on this relationship is not significant; and (3) project commitment negatively moderates the relationship between shame and learning from failure, and the higher the level of project commitment is, the weaker the negative relationship.

### Theoretical Contributions

This paper enriches the research on the antecedent variables of individual learning from failure. Learning from failure has been shown to have significant beneficial effects on individuals (e.g., improving innovation ability; Carmeli and Dothan, 2017) and teams (e.g., improving task performance; Nagayoshi, 2014). In recent years, many scholars have begun to pay attention to the relevant antecedent variables that may promote employees’ learning from failure. An example is the influence of emotional experience brought by failure on learning from failure (Shepherd et al., 2011). However, these studies mostly focus on basic emotions (such as sadness, fear, etc.; Shepherd, 2003) and pay less attention to negative emotions such as shame, which are related to low self-understanding and evaluation. From the perspective of Chinese culture and the impact of shame on individual cognition and behavior, this paper explains why shamed employees find it difficult to learn from failure. In fact, there are many different opinions on the effect of shame, and many scholars have proposed its positive effect on individuals (Wang et al., 2018). Based on the failure situation in Chinese culture, unlike Western culture, “face” and other factors will affect the performance of individuals in multiple aspects, including cognition, emotion, and behavior, after failure events (Wang et al., 2019). Therefore, shame is more likely to have negative effects on individuals. Previous scholars have begun to explore the important influence of Chinese culture on learning from failure in combination with Chinese culture (Wang et al., 2019, 2021). However, comparative studies on Eastern and Western cultures are mostly lacking. Based on the research on shame by Western scholars, this paper shifts the cultural perspective, expands the research conclusions on shame and learning from failure, and expands the application scenarios of the related research on shame.

Based on emotion regulation process theory, we incorporated affective and cognitive variables that might influence the process of “shame-learning from failure.” In fact, in recent years, many scholars have paid attention to the boundary condition variables that may affect the response of learning from failure behaviors. However, scholars mainly focus on self-regulating behaviors (e.g., emotion regulation; Fang He et al., 2018), personality traits related to emotion (e.g., emotional stability; Zhao, 2011), and situational variables (e.g., error management culture; Van Dyck et al., 2005). Dolan (2002) and Phelps (2006) also stated that emotion and cognition have important influences on individual behavioral responses. Therefore, we integrate the two boundary conditions of affective and cognitive traits and integrate the relatively independent studies of Dolan (2002) and Phelps (2006) to integrate affective and cognitive factors into the same framework. First, regarding cognition, learning from failure means that employees acquire and apply knowledge and skills after experiencing failure (Shepherd et al., 2011), and past studies have shown that individual cognitive traits and experiences have significant impacts on the follow-up behavior of failure (Boss and Sims, 2008; Bolinger and Brown, 2015). However, previous studies have paid more attention to the trait factors of individual stability, such as narcissism (Liu et al., 2019), while few studies have focused on variables such as the emotional bond between employees and projects (e.g., project commitment) that involve employees’ attitude and cognition toward projects and work. By studying the project commitment of employees, this paper explores the impact of the emotional connection between employees and projects on the negative emotions after failure on the subsequent learning and provides a new perspective for the exploration of the boundary variables affecting learning from failure.

In addition, regarding emotional recovery, according to grief recovery theory, we explored the important role of an individual’s negative emotion coping orientation and expanded the scope of application of grief recovery theory. Previous studies have shown that loss orientation is beneficial to learning from failure, while restoration orientation is beneficial to emotional recovery but not to learning from failure (Shepherd et al., 2011). Taking emotion coping orientation as a moderating variable, this paper proposes that loss orientation and restoration orientation can alleviate the negative impact of shame on learning from failure through problem processing and emotional recovery, respectively. Most previous scholars combined negative emotion coping orientation with emotions such as grief and lacked the application of other negative emotions (Shepherd et al., 2011). In this paper, negative emotion coping orientation is introduced into the processing and coping of the self-conscious emotion of shame, which also provides a new idea for the subsequent research of scholars; additionally, the application value of negative emotion coping orientation in other failure situations can be further explored.

However, our results show that only restoration orientation moderates the relationship between shame and learning from failure, while loss orientation has no significant moderating effect, which is different from the conclusions of previous studies. Since the restoration orientation focuses more on the recovery of negative emotions, this may have a more significant impact on the negative impact of weakening the sense of shame; on the contrary, because the loss orientation focuses more on handling failure events, it may be more difficult for employees to focus on the recovery of negative emotions, which makes the moderating effect of loss orientation is not significant. Additionally, we believe this is also due to the cross-sectional research design of the study. In fact, as time passes, loss orientation leads to excessive reflection for a long time, thus further producing regret, disappointment, and feelings of anxiety (Shepherd et al., 2011), which may cause cognitive and psychological consumption for employees, making employees unable to concentrate on treating failed events, exploring solutions, and summarizing the experiences of failure. Therefore, empirical sampling methods and other research methods can be used to further study this problem in the future.

### Practical Contributions

The results of our research have important practical implications for managers and organizations. First, enterprises and managers should create a positive organizational atmosphere to reduce employees’ shame after failure events. Examples include telling employees that failure is just a part of the job, normalizing coping with failure, and breaking the corporate system and culture that rewards success and punishes failure. Other examples include supporting employees when facing failure, analyzing failure, handling failure, providing appropriate resources to encourage employees to develop knowledge and skills from failure, and actively cultivating cultural values encouraging learning from failure.

Second, enterprises should improve the emotional management ability of employees through training and other means. Therefore, enterprises can improve employees’ ability to cope with failure and their willingness and ability to learn from failure by developing their restoration orientation so as to alleviate the negative impact of shame on employees’ learning from failure. Specifically, managers can shift employees’ attention by assigning new tasks and making personnel adjustments so as to support employees in acquiring new skills and developing new abilities, which can reduce the interference of shame on employees’ attention and the defensive and aggressive behavior of employees caused by shame.

Finally, enterprises and managers should build a harmonious corporate culture and provide employees with various forms of support (such as employee skill training, career development planning, team activities) to enhance the sense of belonging of employees so as to improve the level of project commitment of employees and their enthusiasm for study and work. Furthermore, enterprises and managers can inform employees of project goals and project values and improve their sense of identity for their work.

### Limitations and Future Research

This paper has drawn relevant conclusions through empirical research, but there are still some limitations as follows. First, the data in this paper are cross-sectional data, which have an impact on the measurement of variables such as emotion coping orientation that may change over time. This may also be a reasonable explanation for the insignificant moderating effect of loss orientation in this study. Therefore, future research can use other methods, such as experience sampling, to collect individual response information at multiple time points and capture the changes in the impacts of loss orientation and restoration orientation on employees’ emotions and learning from failure behaviors. Second, since the scale used in this paper is from the mature scale of foreign countries, there may also be differences in shame as there are differences between Eastern and Western cultural situations. Therefore, future studies can use local scales to verify the results of this study and explore the universality and differences of the results of this model under different cultural situations. Finally, this paper only focuses on the moderating effects of emotion coping orientation and project commitment on the relationship between shame and learning from failure. Whether there are other moderating factors is unknown, and whether there is an interaction between these two factors is also unclear. Therefore, future studies can also further explore the effects of other moderating variables on the model and whether there is an interaction between the moderating variables.

## Data Availability Statement

The raw data supporting the conclusions of this article will be made available by the authors, without undue reservation.

## Ethics Statement

Ethical review and approval was not required for the study on human participants in accordance with the local legislation and institutional requirements. Written informed consent for participation was not required for this study in accordance with the national legislation and the institutional requirements. Written informed consent was not obtained from the individual(s) for the publication of any potentially identifiable images or data included in this article.

## Author Contributions

WW and SS substantially contributed to the conception and the design of the work as well as in the analysis and interpretation of the data. JW, QL, LH, and XC prepared the draft and reviewed it critically. All the authors contributed to the article and approved the submitted version.

## Funding

This research was supported by MOE (Ministry of Education in China) Project of Humanities and Social Sciences (Grant No. 19YJA630082).

## Conflict of Interest

The authors declare that the research was conducted in the absence of any commercial or financial relationships that could be construed as a potential conflict of interest.

## Publisher’s Note

All claims expressed in this article are solely those of the authors and do not necessarily represent those of their affiliated organizations, or those of the publisher, the editors and the reviewers. Any product that may be evaluated in this article, or claim that may be made by its manufacturer, is not guaranteed or endorsed by the publisher.
